# Lapatinib–induced NF-kappaB activation sensitizes triple-negative breast cancer cells to proteasome inhibitors

**DOI:** 10.1186/bcr3575

**Published:** 2013-11-12

**Authors:** Yun-Ju Chen, Ming-Hsin Yeh, Meng-Chieh Yu, Ya-Ling Wei, Wen-Shu Chen, Jhen-Yu Chen, Chih-Yu Shih, Chih-Yen Tu, Chia-Hung Chen, Te-Chun Hsia, Pei-Hsuan Chien, Shu-Hui Liu, Yung-Luen Yu, Wei-Chien Huang

**Affiliations:** 1 Department of Medical Research, E-Da Hospital, Kaohsiung 824, Taiwan; 2 Department of Biological Science & Technology, I-Shou University, Kaohsiung 824, Taiwan; 3 Section of Breast Surgery, China Medical University and Hospital, Taichung 404, Taiwan; 4 Center for Molecular Medicine, China Medical University and Hospital, Taichung 404, Taiwan; 5 Division of Pulmonary and Critical Care Medicine, China Medical University and Hospital, Taichung 404, Taiwan; 6 Department of Internal Medicine, China Medical University and Hospital, Taichung 404, Taiwan; 7 Graduate Institute of Cancer Biology, China Medical University, Taichung 404, Taiwan; 8 Cancer Biology and Drug Discovery, China Medical University, Taichung 404, Taiwan; 9 Department of Respiratory Therapy, China Medical University, Taichung 404, Taiwan; 10 Graduate Institute of Clinical Medical Science, China Medical University, Taichung 404, Taiwan; 11 Department of Public Health, China Medical University, Taichung 404, Taiwan; 12 Department of Pharmacology, College of Medicine, National Taiwan University, Taipei 100, Taiwan; 13 Department of Life Science, National Chung-Hsing University, Taichung 402, Taiwan; 14 Department of Health Care and Social Work, Yu Da University of Science and Technology, Miaoli, Taiwan; 15 Department of Biotechnology, Asia University, Taichung 413, Taiwan

## Abstract

**Introduction:**

Triple-negative breast cancer (TNBC), a subtype of breast cancer with negative expressions of estrogen receptor, progesterone receptor, and human epidermal growth factor receptor 2 (HER2), is frequently diagnosed in younger women and has poor prognosis for disease-free and overall survival. Due to the lack of known oncogenic drivers for TNBC proliferation, clinical benefit from currently available targeted therapies is limited, and new therapeutic strategies are urgently needed.

**Methods:**

Triple-negative breast cancer cell lines were treated with proteasome inhibitors in combination with lapatinib (a dual epidermal growth factor receptor (EGFR)/HER2 tyrosine kinase inhibitor). Their *in vitro* and *in vivo* viability was examined by MTT assay, clonogenic analysis, and orthotopic xenograft mice model. Luciferase reporter gene, immunoblot, and RT-qPCR, immunoprecipitation assays were used to investigate the molecular mechanisms of action.

**Results:**

Our data showed that nuclear factor (NF)-κB activation was elicited by lapatinib, independent of EGFR/HER2 inhibition, in TNBCs. Lapatinib-induced constitutive activation of NF-κB involved Src family kinase (SFK)-dependent p65 and IκBα phosphorylations, and rendered these cells more vulnerable to NF-κB inhibition by p65 small hairpin RNA. Lapatinib but not other EGFR inhibitors synergized the anti-tumor activity of proteasome inhibitors both *in vitro* and *in vivo*. Our results suggest that treatment of TNBCs with lapatinib may enhance their oncogene addiction to NF-κB, and thus augment the anti-tumor activity of proteasome inhibitors.

**Conclusions:**

These findings suggest that combination therapy of a proteasome inhibitor with lapatinib may benefit TNBC patients.

## Introduction

Triple-negative breast cancer (TNBC), defined by the lack of expression of estrogen, progesterone, and epidermal growth factor receptor/human epidermal growth factor receptor 2 (ERBB2/HER2) receptors [1], represents 15% to 20% of all breast cancer cases [2] and occurs in young premenopausal women with a higher frequency [3]. TNBC is commonly associated with basal-like phenotype and characterized by high histological grade, preference for brain or lung metastasis and aggressive behavior with shorter time to recurrence and death [1,3]. Patients with this subtype usually have a worse clinical outcome [2]. In addition to an intricate relationship with basal-like breast carcinomas, TNBC is gaining attention due to its lack of effective tailored therapies. Chemotherapy is the systemic therapy currently available for TNBC, but no standard regimen is recommended. Some TNBC tumors are sensitive to paclitaxel-containing and doxorubicin-containing chemotherapies [4]. However, TNBC patients became rapidly chemoresistant and frequently relapsed, and showed a worse prognosis [5,6]. New therapeutic strategies are urgently needed.

NF-κB (nuclear factor kappa-light-chain-enhancer of activated B cells) is a family of transcription factors involved in the regulation of immune responses and inflammation, and plays a major role in tumorigenesis of many cancer types [7,8]. It is restricted to the cytoplasm by binding with inhibitory IκB proteins. In response to stimulations, IκB kinase (IKK) complex is activated to phosphorylate IκB proteins. The phosphorylated IκB proteins are then ubiquitinated and degraded by 26S proteasome [9], leading to NF-κB nuclear translocation. NF-κB controls the expressions of several pro-tumorigenic genes which are associated with angiogenesis, apoptosis, invasion, migration, and cell survival [10,11]. Aberrant activation of NF-κB also enhances resistance to chemotherapy in cancer cells [12]. Inactivation of NF-κB through blocking IκB degradation by bortezomib, a proteasome inhibitor, has shown clinical benefits for the treatment of hematological malignancies [13]. Although NF-κB activation and overexpression of its target genes have been observed in TNBC tumors [14,15], bortezomib showed limited clinical benefits in phase II trials [16]. These disappointing results suggest that the survival of TNBC may only be partially addicted to NF-κB. Therefore, new strategies making TNBC more addicted to NF-κB activity may be able to improve the therapeutic efficacy of proteasomal inhibitors.

Lapatinib (GW572016, Tykerb), a dual EGFR and HER2 tyrosine kinase inhibitor (TKI), has been approved for trastuzumab-resistant HER2-positive advanced breast cancer patients, [17]. However, acquired resistance still occurred within six to twelve months after initial treatment [18]. The elevation of NF-κB activity was found in lapatinib-treated HER2-positive breast cancer cells [19,20], and targeting RelA (p65) protein expression enhanced the lapatinib-induced apoptosis [20]. Lapatinib has recently been found to up-regulate the gene expression of proapoptotic TRAIL death receptors DR4 and DR5 [21]. Our recent study also showed that lapatinib can induce the NF-κB-targeted gene COX-2 in a HER2/EGFR-independent manner [22]. These observations raise the possibility that lapatinib may increase NF-κB activity independently of targeting EGFR and HER2.

In this study, we demonstrated that lapatinib, but not specific EGFR inhibitors gefitinib and erlotinib, can induce the phosphorylation and nuclear translocation of p65 and the subsequent expression of NF-κB target genes in both HER2-positive and TNBC cells. We further revealed the involvement of Src family kinase (SFK)-dependent p65 and IκBα phosphorylations in this event. Although lapatinib or bortezomib alone did not elicit the expected clinical benefits for TNBC, co-treatment can enhance the anti-tumor activity of bortezomib by increasing the oncogenic addiction of these cancer cells to NF-κB. These findings not only decipher the molecular mechanisms of lapatinib-induced NF-κB activation, but also suggest remarkable therapeutic benefits with combination of bortezomib and lapatinib in TNBC patients.

## Methods

### Cell lines and reagents

All cancer cell lines were purchased from the American Type Culture Collection (ATCC – Manassas, VA, USA) and maintained in a humidified 5% CO2 incubator at 37°C. SkBr3, BT474, MDA-MB-231, MDA-MB-468 and HBL100 were cultured in (Dulbecco’s) modified Eagle’s medium ((D)MEM)/F12. HS-578 T was maintained in Roswell Park Memorial Institute medium (RPMI). All the media were supplemented with 10% FBS, 100 unit/ml penicillin and 100 mg/ml streptomycin. To establish lapatinib-, erlotinib-, and gefitinib-selected cells, cancer cells were treated with increasing concentrations of lapatinib, erlotinib, or gefitinib up to 1 μM. *p65* shRNA clones were purchased from the National RNAi Core Facility at Academia Sinica (Taipei, Taiwan).

### Protein extraction and immunoblot

For total cell lysates, cells were washed with ice-cold PBS one time and lysed in RIPA buffer (20 mM Tris–HCl, pH7.4, 150 mM NaCl, 1% NP-40, 1% sodium deoxycholate, 1 mM ethylenediaminetetraacetic acid (EDTA) and 1 mM ethylene glycol tetraacetic acid (EGTA)). For subcellular fractionation, the methods were done as previously described. Protease inhibitors and phosphatase inhibitors cocktails were added in the RIPA buffer. Proteins were separated by SDS-PAGE, transferred to a polyvinylidene fluoride (PVDF) membrane and blotted with indicated antibodies.

### Immunofluorescence staining

Cells were grown on gelatin cover slips and fixed at day 2 with 4% paraformaldehyde in PBS for 15 minutes. For immunofluorescence staining, cells were next treated with 0.5% Triton X-100 in PBS for 15 minutes and blocked with 10% BSA in PBS for 1 hour followed by incubation with anti-p65 antibody at 4°C overnight. After incubation with horseradish peroxidase (HRP)-labeled secondary antibody, cells were further stained with the nucleic acid stain, diamidino-2-phenylindole (DAPI) (Invitrogen, Carlsbad, CA, USA), and mounted with ProLong Gold antifade mounting reagent (Invitrogen).

### Microarray analysis and ingenuity pathway analysis

Total RNA was extracted by Trizol® Reagent (Invitrogen) according to the instruction manual. RNA was quantified at OD260 nm by using a ND-1000 spectrophotometer (Nanodrop Technology, Wilmington, Delaware USA) and qualitated by using a Bioanalyzer 2100 (Agilent Technology, Santa Clara, California USA) with RNA 6000 nano labchip kit (Agilent Technologies). Total RNA (0.5 mg) was amplified by a Quick-Amp Labeling kit (Agilent Technologies) and labeled with Cy3 or Cy5 (CyDye, PerkinElmer, Waltham, Massachusetts USA) during the *in vitro* transcription process. CyDye-labled cRNA (0.825 mg) was fragmented to an average size of about 50 to 100 nucleotides by incubation with fragmentation buffer at 60°C for 30 minutes. Correspondingly fragmented labeled cRNA was then pooled and hybridized to Agilent Human Whole Genome Oligo 4 × 44 K Microarray (Agilent Technologies) at 60°C for 17 hours. After washing and drying by nitrogen gun blowing, microarrays were scanned with an Agilent microarray scanner (Agilent Technologies) at 535 nm for Cy3 and 625 nm for Cy5. Scanned images were analyzed by Feature extraction 9.5.3 software (Agilent Technologies), an image analysis and normalization software was used to quantify signal and background intensity for each feature, and the data substantially normalized using the rank-consistency-filtering LOWESS method. The data have been deposited in NCBI’s Gene Expression Omnibus and are accessible through GEO Series accession number GSE51889 [23].

NF-κB-targeted gene expressions were overlaid onto a global molecular network developed from information contained in the Ingenuity Pathways Knowledge Base (IPA Ingenuity Systems [24]). The network of these NF-κB-targeted genes was then algorithmically generated based on their connectivity and the molecular relationships between these genes/gene products were presented graphically. The NF-κB-targeted genes or gene products are represented as nodes, and the biological relationship between two nodes is represented as an edge (line). All edges are supported by at least one reference from the literature, from a textbook, or from canonical information stored in the Ingenuity Pathways Knowledge Base. The intensity of the node color indicates the degree of positive (red) or negative (green) correlation. Nodes are displayed using various shapes that represent the functional class of the gene product. Edges are displayed with various labels that describe the nature of the relationship between the nodes.

### RNA extraction and quantitative reverse transcription PCR

Total RNA was extracted by Trizol™ reagent (Roche, Basel, Switzerland) and converted into cDNA with M-MLV reverse transcriptase (Invitrogen). Synthesized cDNA was used as the template for SYBR qPCR, and changes in gene expression level were normalized to *GAPDH* in respective cDNA samples. Primers used were as described below: 5′-CGGACTGCCCTTCACCTCGC-3′ (IκBα, forward), 5′-GTATCCGGGTGCTTGGGCGG-3′ (IκBα, reverse), 5′-CCCCCGACTGGACGAGAGGG-3′ (ICAM1, forward), 5′-TGAGTGCTCCTGGCCCGACA-3′ (ICAM1, reverse), 5′-CTTCAGGCAGGCCGCGTCAG-3′ (IL-1β, forward), 5′-TGCTGTGAGTCCCGGAGCGT-3′ (IL-1β, reverse), 5′-TGGCACCTGACCGGAGCATGTA-3′ (TRAF1, forward), 5′-CAACAGGTGGCCTCTGGGCTGT -3′ (TRAF1, reverse), and 5′GCTTAAACAGGAGCATCCTGA-3′ (COX-2, forward), and 5′-GGGTAATTCCATGTTCCAGC-3′ (COX-2, reverse).

### 3-(4,5-dimethylthiazol-2-yl)-2,5-diphenyltetrazolium bromide (MTT) cell viability assay

*In vitro* cell viability was measured using an MTT colorimetric assay. Cells were trypsinized to seed at a density of 5 × 10^3^ to 1 × 10^4^ cells/well in 96-well plates. After various treatments and culturing periods, the culture medium was removed and 100 μL of serum-free medium with 5 mg/mL MTT solution (Sigma, St. Louis, MO, USA), 25 μL/well, was added and the cells incubated for three hours. Finally, 100 μL of dimethyl sulfoxide (DMSO) was added to lyse the cells and the O.D.550 wavelength was detected by ELISA reader.

### Clonogenic assay

For clonogenic assay, cells were plated at 5 × 10^3^ to 1 × 10^4^ cells/well in 6-well plates and pretreated with 1 μM lapatinib or gefitinib for three days followed by addition of MG132 or bortezomib. Colonies were fixed and stained with 30% ethanol containing 1% crystal violet.

### Reporter gene assay

Cells were seeded at 2 × 10^5^ cells/well in 12-well plates and transfected with the indicated plasmids in each experiment, including *IL-1 β* promoter-luciferase plasmids containing NF-κB binding sites. Twenty-four hours after transfection, the luciferase activities in cell lysates were determined by the Luciferase Assay System (Promega, Madison, WI, USA) and normalized to β-galactosiadase activities.

### Tumor xenograft mouse model

All animal experiments were performed in accordance with a protocol approved by the Institutional Animal Care and Use Committee of China Medical University and Hospital (No. 100-61-N). Female severe combined immunodeficient (SCID) mice at 4 to 6 weeks of age were used in the orthotopic tumor-xenograft model. MDA-MB-231 cells (6 × 10^6^ cells/mouse; re-suspended in a 1:1 mixture of PBS and growth factor–reduced Matrigel (BD Biosciences, San Jose, CA, USA) in a total volume of 50 μL) were injected into the mammary fat pads of SCID mice, and the tumor size was measured regularly. Once the tumor size reached 100 mm^3^, mice were treated orally with saline, lapatinib (20 mg/kg), bortezomib (0.02 mg/kg), or a combination of lapatinib (20 mg/kg) and bortezomib (0.02 mg/kg) every day. One month later, all mice were sacrificed, and tumors were resected and their size was measured. The tumor growth rates were analyzed by measuring the tumor length (L) and width (W) with calipers and by calculating the volume with the formula LW^2^/2.

### Immunohistochemical Staining

Five-micron thick paraffin-embedded tissue sections were deparaffinized and rehydrated. The tissue sections were incubated for two hours with mouse monoclonal anti-human p65 and anti-Bax antibodies (100 dilution, Santa Cruz, Dallas, Texas USA). After washing to remove unbound primary antibody, sections were treated with a dextran polymer backbone conjugated to secondary antibodies and labeled with HRP according to the manufacturer’s instructions (DAKO Envision system for mouse and rabbit primary antibodies, DAKO Corporation, Carpinteria, CA, USA) for 30 minutes. Tissue sections were incubated in the chromogenic peroxidase substrate, diaminobenzidine, for five minutes. The specificity of labeling by this procedure was verified by negative control reactions using buffer to replace the primary antibody and isotype-specific immunoglobulin G (IgG).

### Statistical analysis

All results are presented as the mean ± SD. A two-tailed Student’s *t*-test was used to calculate the statistical significance between the groups. The tumor volume was analyzed by a two-sided *t*-test.

## Results

### Lapatinib induces NF-κB activation independently of EGFR/HER2 inhibiton

**Figure 1:**
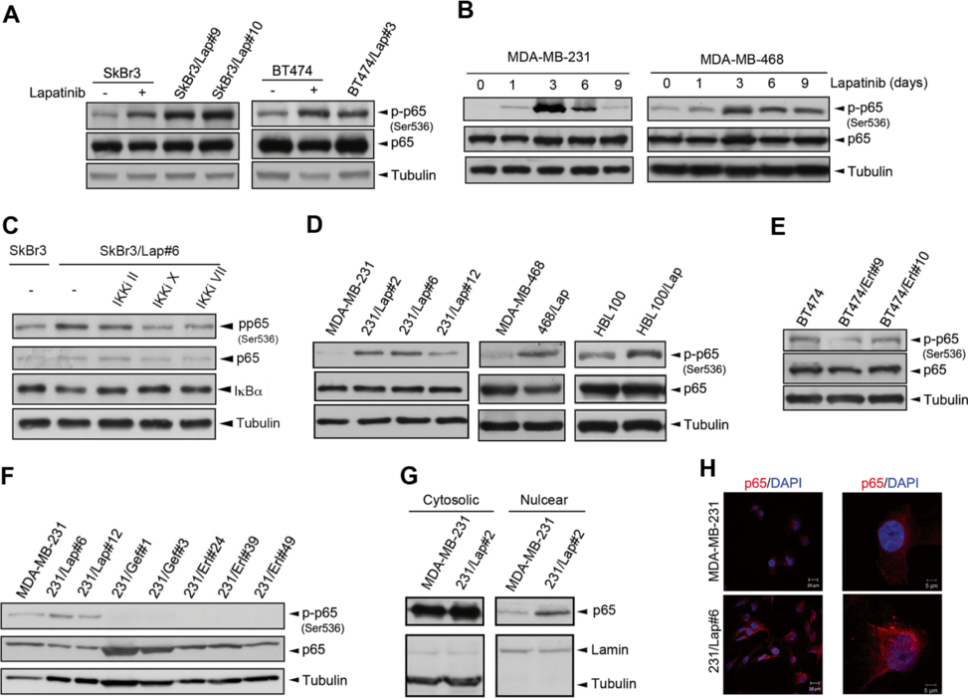
**Lapatinib induces NF-κB activation in an off-target manner. (A-F)** Lapatinib induces p65 phosphorylation in both HER2-positive and triple-negative breast cancer cells. HER2-postive SkBr3 and BT474 breast cancer cells were treated with or without 1 μM lapatinib for two days, and SkBr3/Lap and BT474/Lap cells were cultured in the presence of 1 μM lapatinib **(A)**. Triple-negative MDA-MB-231 and MDA-MB-468 breast cancer cells were treated with 1 μM lapatinib for the indicated days **(B)**. SkBr3/Lap#6 cells were treated with 10 μM IKK inhibitor II, 20 μM IKK inhibitor X, or 5 μM IKK inhibitor VII for two days **(C)**. Lapatinib-selected clones established from triple-negative MDA-MB-231, MDA-MB-468, and HBL-100 cell lines were cultured in the presence of 1 μM lapatinib **(D)**. Erlotinib- and gefitinib-selected clones established from BT474 **(E)** and MDA-MB-231 cells **(F)** were cultured in the presence of 1 μM respective drugs. Total lysates prepared from these cells were subjected to Western blot analysis with the indicated antibodies for NF-κB phosphorylation and expression. **(G, H)** Lapatinib treatment induces p65 nuclear translocation. MDA-MB-231 and 231/Lap#2 cells were subjected to nuclear/cytoplasmic fractionation followed by Western blot analysis with the indicated antibodies. Lamin and tubulin function as control markers for nuclear and cytosolic extraction, respectively **(G)**. MDA-MB-231 and 231/Lap#6 cells were fixed followed by staining with anti-p65 antibody and DAPI (fluorescent dye for nucleus staining) in confocal microscope analysis **(H)**. DAPI, diamidino-2-phenylindole; IKK, IKB kinase; HER2, human epidermal growth factor receptor 2.

To address whether lapatinib can induce NF-κB activation in both HER2-positive and HER2-negative breast cancer cells, its effect on p65 Ser536 phosphorylation, a critical IKK complex-mediated modification for NF-κB activity [25], was examined. Lapatinib markedly induced p65 Ser536 phosphorylation in HER2-positive SkBr3 and BT474 breast cancer cell lines (Figure 1A) and in HER2-negative MDA-MB-231 and MDA-MB-468 cell lines (Figure 1B). To further examine the activation of NF-κB after long-term treatment with lapatinib acquired resistant clones of SkBr3 and BT474 cell lines, named SkBr3/Lap and BT474/Lap respectively, were established by chronic treatment with increasing concerntrations of lapatinib. p65 Ser536 phosphorylation remained higher in both SkBr3/Lap and BT474/Lap clones compared with their parent cells (Figure 1A) and IKK inhibitors suppressed this phosphorylation in SkBr3/Lap#6 cells (Figure 1C), supporting that lapatinib resistance induced activation of the IKK/NF-κB axis. To examine whether this event occurs in HER2-negative breast cancer cells, lapatinib-selected clones from triple-negative MDA-MB-231, MDA-MB-468, and HBL100 cell lines, named 231/Lap, 468/Lap, and HBL100/Lap respectively, were also established. The induction of p65 phosphorylation by lapatinib was also observed in these lapatinib-selected TNBC clones (Figure 1D) and this phosphorylation in SkBr3/Lap#6, BT474/Lap#3, and 231/Lap#6 cells was reduced once lapatinib was withdrawn from the culture medium (Additional file 1: Figure S1), suggesting that lapatinib activates NF-κB independently of HER2 inhibition. In addition, EGFR specific inhibitors gefitinib (Gef)- or erlotinib (Erl)-selected clones from BT474 and MDA-MB-231 cells were established. However, these two EGFR inhibitors did not increase p65 Ser536 phosphorylation. These results suggest that inhibition of EGFR and HER2 was not involved in the lapatinib-induced NF-κB activation.

In addition to phosphorylation at Ser536, p65 phosphorylations at other residues, such as Ser276 and Ser529, are also critical for the NF-κB activation in response to various stimuli [26,27]. However, p65 Ser276 phosphorylation (Additional file 1: Figure S2A and S2B) and Ser529 (Additional file 1: Figure S2C and S2D) were suppressed rather than increased in the SkBr3/Lap, BT474/Lap and 231/Lap clones. To further confirm NF-κB activation, the nuclear localization of p65 in these lapatinib-treated cells was examined. Our data showed that the nuclear p65 level was significantly higher in 231/Lap#2 clone than in the MDA-MB-231 cells (Figure 1G), and this event was observed in immunofluorescence staining/confocal microscope analysis (Figure 1H). All these results indicated that lapatinib possesses off-target activity to induce p65 phosphorylation and nuclear translocation in both HER2-positive and TNBC cells.

**Figure 2:**
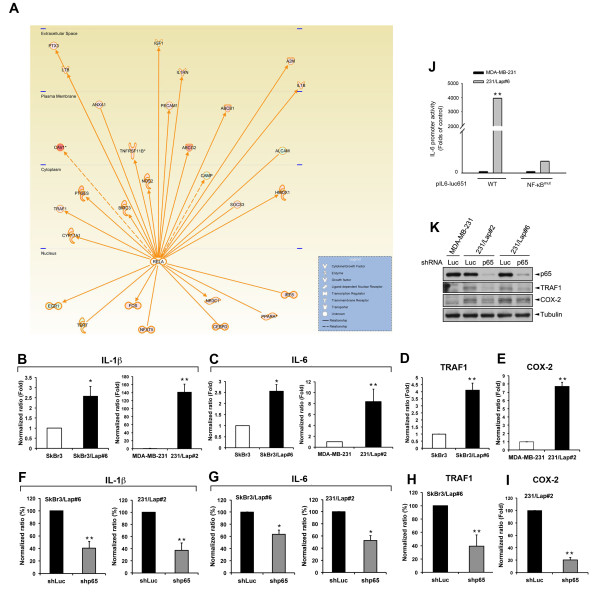
**Expressions of NF-κB target genes are elevated by lapatinib in both HER2-positive and –negative breast cancer cells. (A)** Total RNA extracted from two pairs of parental and lapatinib-resistant cells (SkBr3 and SkBr3/Lap#6; BT474 and BT474/Lap#3) was subjected to reverse transcription followed by cDNA microarray analysis. Gene expressions of NF-κB target gene with more than two folds change were analyzed with Ingenuity Pathway Analysis (IPA). **(B-E)** Total RNA extracted from SkBr3, MDA-MB-231, and their lapatinib-selected clones was subjected to RT-qPCR analysis for mRNA levels of IL-1β **(B)**, IL-6 **(C)**, TRAF-1 **(D)** and COX-2 **(E)**. **(F-I)** SkBr3/Lap#6 and 231/Lap#2 cells were infected with lentiviral shRNA against luciferase (shLuc) or p65 (shp65) for three days, and then total RNA was extracted and subjected to RT-qPCR analysis for mRNA levels of IL-1β **(F)**, IL-6 **(G)**, TRAF-1 **(H)** and COX-2 **(I)**. **(J)** MDA-MB-231 and 231/Lap#6 cells were transiently transfected with the luciferase reporter gene of *IL-6* promoter containing wild-type (WT) or mutation of NF-κB-binding sites (NF-κB^mut^). The promoter activity of *IL-6* was normalized with β-galactosidase activity. **(K)** MDA-MB-231 and 231/Lap cells were infected with lentivirus of shRNA against luciferase (shLuc) or p65 (shp65) for three days, and then whole protein lysates were prepared and subjected to Western blot analysis with antibodies against indicated molecules. shRNA, short hairpin RNA.

We next investigated whether the lapatinib-activated NF-κB can mediate its downstream gene expressions. cDNA microarray analysis was employed to profile gene expressions that were upregulated more than two folds in lapatinib-resistant SkBr3 and BT474 cells (Additional file 2: Table S1). Then, this data were analyzed with Ingenuity Pathway Analysis (IPA) to identify which genes are regulated by NF-κB in response to lapatinib treatment. The result showed 24 genes upregulated by NF-κB (Figure 2A), and some well-documented NF-κB-target genes were further validated. The mRNA levels of *IL-1β*, *IL-6* and *TRAF1* in SkBr3/Lap#6 cells were higher than those in SkBr3 cells (Figure 2B-D) and were reduced by p65 shRNA (Figure 2F-H). Similarly, *IL-1β, IL-6* and *COX-2* transcripts were also induced in 231/Lap#2 cells (Figure 2B, C, and E) and were inhibited by p65 shRNA (Figure 2F, G, and I). *IL-1β* and *IL-6* transcripts were also increased by short-term treatment with lapatinib in SkBr3 and MDA-MB-231 cells (Additional file 1: Figure S3A and S3B) and were reduced in response to the lapatinib withdrawal in SkBr3/Lap#6 and 231/Lap#2 cells (Additional file 1: Figure S3C and S3D). *IL-6* promoter activity was dramatically higher in 231/Lap#6 cells than in the parental cells. Mutation of the NF-κB-binding site almost abolished this effect (Figure 2J), indicating that lapatinib-activated NF-κB mediated the gene transcription in response to lapatinib treatment. The protein levels of COX-2 and TRAF1 were also increased in both 231/Lap#2 and 231/Lap#6 cells and were suppressed by p65 shRNA (Figure 2K). These results show that lapatinib treatment not only activates NF-κB but also subsequently induces various NF-κB-targeted gene expressions.

### Induction of NF-κB by lapatinib involves rapid IκBα turnover and tyrosine phosphorylation

**Figure 3:**
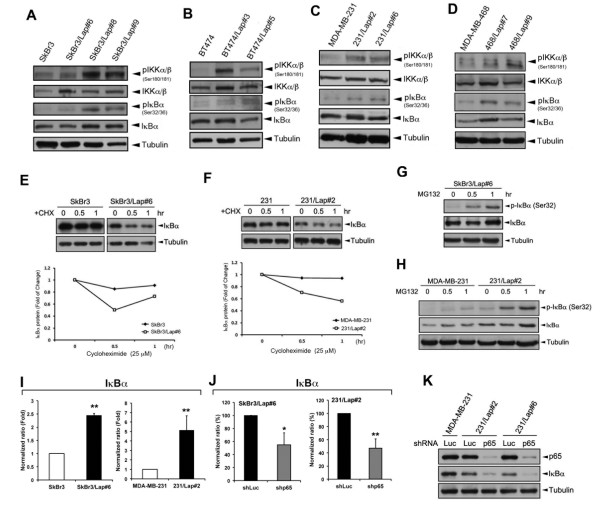
**Activation of NF-κB by lapatinib involves rapid IκBα turnover. (A-D)** Whole cell lysates from parental and lapatinib-selected cells of HER2-positive SkBr3 **(A)** and BT474 **(B)** cell lines and triple–negative MDA-MB-231 **(C)** and MDA-MB-468 **(D)** cell lines breast cancer cells were harvested and subjected to Western blot analysis with specific antibodies for phosphorylation and protein expression of IKKα/β, IκBα, and tubulin. **(E,F)** SkBr3 **(E)**, MDA-MB-231 **(F)**, and their lapatinib-treated clones were treated with 25 μM cycloheximide for 0, 0.5 and 1 hours. The protein expression of IκBα was examined by Western blot analysis and quantified. **(G,H)** SkBr3/Lap **(G)**, MDA-MB-231, and 231/Lap#2 **(H)** cells were treated with 1 μM MG-132 for 0, 0.5 and 1 hours. The protein expression and phosphorylation of IκBα were examined by Western blot analysis. **(I,J)** Total RNA was extracted from parental and lapatinib-resistant clones of SkBr3 and MDA-MB-231 cells **(I)** and from SkBr3/Lap#6 and 231/Lap#2 cells infected with lentivirus of Luc or p65 shRNA for three days **(J)**. The expression of IκBα mRNA was analyzed by RT-qPCR. **(K)** MDA-MB-231 and 231/Lap cells were infected with lentivirus of Luc or p65 shRNA for three days. The protein expression of IκBα was analyzed by Western blot analysis. shRNA, short hairpin RNA.

Although activation of the IKK complex and its downstream IκBα Ser32/36 phosphorylations were found in SkBr3/Lap and BT474/Lap clones, reduction of IκBα was not seen (Figure 3A and B). Similar phenomena were also observed in lapatinib-treated clones of MDA-MB-231 and MDA-MB-468 (Figure 3C and D). Therefore, whether IκBα degradation was involved in lapatinib-induced NF-κB activation was further investigated. Since IκBα is also a downstream target gene of NF-κB, we hypothesized that lapatinib still can induce IκBα degradation but the protein level of IκBα was retained at a steady state in lapatinib-treated clones due to *de novo* synthesis. To prove this hypothesis, we treated cells with cycloheximide to inhibit protein biosynthesis and monitored the IκBα level by Western blot analysis. In parental SkBr3 (Figure 3E, left) and MDA-MB-231 (Figure 3F, left) cells, IκBα remained unchanged after treatment with cycloheximide; however, in SkBr3/Lap (Figure 3E, right) and 231/Lap (Figure 3F, right) cells, IκBα declined quickly in the presence of cycloheximide. Furthermore, the IκBα level and Ser32/36 phosphorylations in SkBr3/Lap#6 (Figure 3G) and in 231/Lap#2 (Figure 3H) cells were also elevated when cells were treated with proteasome inhibitor MG132. These results suggest that IκBα protein was continuously degraded but was also re-synthesized simultaneously back to the steady state in response to lapatinib treatment. In support to this notion, an increase in *IκBα* mRNA level was found in both SkBr3/Lap and 231/Lap clones (Figure 3I), and silencing of p65 expression can down-regulate the transcripts (Figure 3J) and protein expression (Figure 3K) of IκBα. These results suggest that lapatinib-induced NF-κB activation still involved IκBα protein degradation, and the unchanged IκBα level may be due to the elevation of *de novo* gene expression.

**Figure 4:**
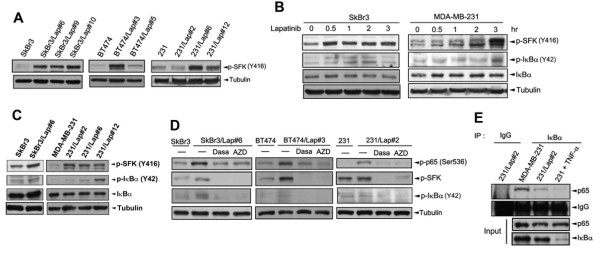
**SFK activation contributes to induction of NF-κB by lapatinib. (A)** Whole cell lysates of SkBr3, BT474, MDA-MB-231 and their lapatinib-selected clones were collected followed by Western blot analysis with anti-phospho-SFK and anti-tubulin antibodies. **(B)** SkBr3 and MDA-MB-231 cells were treated with lapatinib for the indicated time points. Phosphorylation of p-SFK and p-IκBα as well as IκBα and tubulin protein expression were detected by Western blot analysis. **(C)** Whole cell lysates from SkBr3, MDA-MB-231 cells and their lapatinib-treated clones were harvested and subjected to Western blot analysis with specific antibodies for phosphorylation and/or expression of SFK and IκBα. **(D)** SkBr3, BT474, MDA-MB-231 and their lapatinib-selected clones were treated with the SFK inhibitors, dasatinib (Dasa) or AZD0530 (AZD). Total lysates were prepared and subjected to Western blot analysis with anti-phospho-p65 (Ser536), anti-phospho-SFK and anti-tubulin antibodies. **(E)** IκBα protein from total lysates of MDA-MB-231 and 231/Lap cells was immunoprecipitated followed by Western blot analysis with anti-p65 antibody for IκBα/p65 protein interaction. SFK, Src family kinase.

In addition to the IκBα phosphorylations at Ser32/36 by the IKK complex, phosphorylation at Tyr42 by Src can also lead to NF-κB activation by reducing the protein interaction between p65 and IκBα [28,29]. c-Src/Lck is activated in response to stresses, such as hypoxia and X-rays, and causes Tyr42 phosphorylation of IκBα without its protein degradation [28,29]. To clarify whether SFK also participates in the NF-κB activation in lapatinib-resistant cells, the SFK activity in resistant cells was examined. Our data revealed that tyrosine phosphorylation of SFK is increased in lapatinib-treated clones of SkBr3, BT474, and MDA-MB-231 (231) cells (Figure 4A). The phosphorylation of SFK was increased by lapatinib treatment for a few days in the parental BT474 cells (Additional file 1: Figure S4A), but was reduced in lapatinib-resistant clones of SkBr3 and MDA-MB-231 cells upon removal of lapatinib from the culture medium (Additional file 1: Figure S4B). Short-term treatment with lapatinib can also induce IκBα Tyr42 phosphorylation in both HER2-positive SkBr3 and HER2-negative MDA-MB-231 cells (Figure 4B and Additional file 1: Figure S4C), which was also observed in SkBr3/Lap#6 and 231/Lap clones (Figure 4C). Both p65 Ser536 phosphorylation and IκBα Tyr42 phosphorylation in lapatinib-treated SkBr3, BT474 and MDA-MB-231 cells were suppressed by Src inhibitors, including dasatinib (Dasa), AZD0530 (AZD) and PP2 (Figure 4D), suggesting that the lapatinib-induced NF-κB activation is Src-dependent. Anti-IκBα immunoprecipitates were immunoblotted with anti-p65 antibody to further confirm the involvement of Tyr42 phosphorylation of IκBα in NF-κB activation, and the interaction between IκBα and p65 was reduced in 231/Lap cells compared to parental cells (Figure 4E). These results suggest that Src-dependent Tyr42 phosphorylation of IκBα may render the *de novo* IκBα unable to feedback repress NF-κB activity.

### Activated NF-κB becomes the Achilles’ heel in lapatinib-treated HER2-negative breast cancer cells

**Figure 5:**
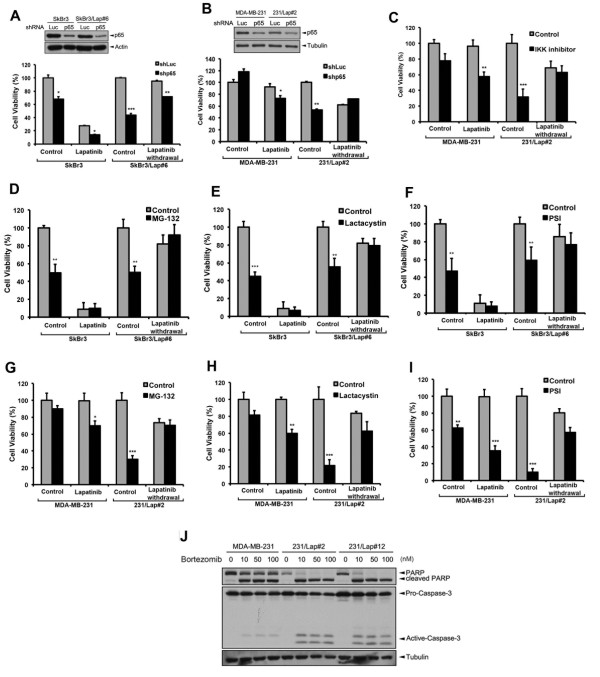
**NF-κB functions as a critical regulator for cell survival in response to lapatinib treatment. (A,B)** SkBr3 **(A)**, MDA-MB-231 **(B)**, and their lapatinib-treated derivatives were infected with lentivirus of Luc or p65 shRNA in the presence or absence of 1 μM lapatinib for three days. Cell viability and p65 protein expression were determined by MTT and Western blot analyses, respectively. **(C)** MDA-MB-231 and 231/Lap cells were treated with 10 μM IKKα inhibitor in the presence or absence of 1 μM lapatinib for 24 hours. The cell viability was determined by MTT assay. **(D-I)** Parental and lapatinib-selected cells of SkBr3 **(D-F)** and MDA-MB-231 **(G-I)** cells were treated with various doses of proteasome inhibitors, including MG-132 **(D and G)**, lactacystin **(E and H)** and proteasome inhibitor I **(F and I)** in the presence or absence of lapatinib for 24 hours and then subjected to MTT assay. **(J)** MDA-MB-231 cells and their lapatinib-treated clones were treated with or without the indicated concentrations of bortezomib for 48 hours. Two apoptotic markers, PARP and caspase 3 cleavages, were then examined by Western blot analysis with anti-PARP and anti-caspase 3 specific antibodies. *, *P* <0.05; **, *P* <0.01; ***, *P* <0.001. IKK, IKB kinase; MTT, 3-(4,5-dimethylthiazol-2-yl)-2,5-diphenyltetrazolium bromide; PARP, poly ADP ribose polymerase.

Since NF-κB is activated in both HER2-positive and HER2-negative breast cancer cells in response to lapatinib treatment, we next examined the effect of p65 shRNA on the cell viability of SkBr3 and MDA-MB-231 cells and their lapatinib-resistant clones in both the presence and absence of lapatinib. Although silencing of p65 reduced cell viability in SkBr3 but not in MDA-MB-231 cells, treatment with lapatinib enhanced their sensitivity to p65 shRNA in both cell lines (Figure 5A and B). The cell viability inhibition by p65 shRNA was more dramatic in both SkBr3/Lap#6 and 231/Lap#2 cells in the presence of lapatinib, but the suppression was attenuated after lapatinib withdrawal (Figure 5A and B). Similarly, lapatinib also enhanced the sensitivity of MDA-MB-231 cells to IKKα inhibitor (Figure 5C). The 231/Lap#2 cells also showed greater sensitivity to IKK inhibitor than their parental cells, and this effect was diminished upon lapatinib withdrawal from the culture medium (Figure 5C). These results suggest a critical role of IKK/NF-κB activation in the lapatinib-resistance of both HER2-positive and TNBC cells. Prevention of IκBα degradation via inhibiting proteasome activity efficiently impairs NF-κB activation. Treatment with proteasome inhibitors including MG-132, Lactacystin and Proteasome inhibitor I (PSI) did not further enhance the anti-cancer activity of lapatinib in SkBr3 cells due to the high sensitivity of this cell line to lapatinib (Figure 5D-F). Nevertheless, these proteasome inhibitors circumvented the lapatinib resistance in SkBr3/Lap#6 cells but this effect was not observed after lapatinib was removed from the culture medium (Figure 5D-F). These results suggest that inhibition of NF-κB activation by proteasome inhibitors may overcome the acquired lapatinib resistance in HER2-positive breast cancer cells. Our data further showed that the cell viability of MDA-MB-231 cells was little affected by proteasome inhibitors, but the inhibition was significantly enhanced by lapatinib (Figure 5G-I). Interestingly, proteasome inhibitors dramatically inhibited the cell viability of 231/Lap#2 cells in the presence of lapatinib, and lapatinib withdrawal diminished the sensitivity (Figure 5G-I). Poly ADP ribose polymerase (PARP) and caspase-3 protein cleavages induced by the proteasome inhibitor bortezomib were also obviously enhanced in 231/Lap clones (Figure 5J). These results not only suggest that lapatinib can enhance the oncogenic addiction of TNBC cells to NF-κB, but also imply that co-treatment of lapatinib may enhance their sensitivity to proteasome inhibitors.

### Lapatinib treatment sensitizes TNBCs to proteasome inhibitors

**Figure 6:**
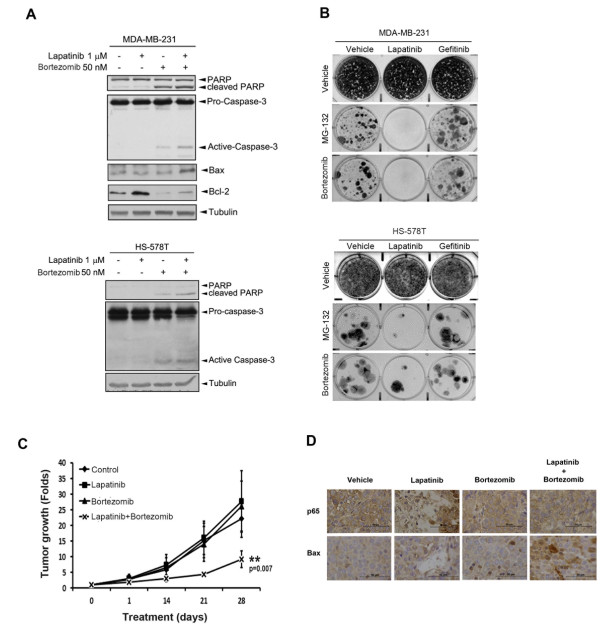
**Lapatinib significantly enhances the anti-tumor activity of proteasome inhibitors *in vitro *and *in vivo*. (A)** Triple-negative MDA-MB-231 and HS-578 T breast cancer cells were pre-treated with 1 μM lapatinib for three days followed by addition or not of 50 nM bortezomib for 48 hours. Protein cleavage of PARP and caspase 3 were then examined by Western blot analysis with anti-PARP and anti-caspase 3 specific antibodies. **(B)** Triple-negative MDA-MB-231 and HS-578 T breast cancer cells were pre-treated with 1 μM lapatinib or 1 μM gefitinib for three days followed by addition or not of 0.3 μM MG-132 or 50 nM bortezomib for seven days. The cell viability was determined by crystal violet staining. **(C,D)** MDA-MB-231 cells were injected into the mammary fat pad of SCID mice followed by treatment with lapatinib, bortezomib or lapatinib plus bortezomib, respectively. The growth rate of xenograft tumor was determined by measurement of tumor size **(C)**. The expression of p65 and apoptotic protein Bax in the xenograft tumor in response to these treatments was examined by immunohistochemical staining with anti-p65 and anti-Bax specific antibodies **(D)**. PARP, poly ADP ribose polymerase; SCID, severe combined immunodeficiency.

Next, we tested our hypothesis that lapatinib can enhance or switch the oncogenic addiction of TNBC cells to NF-κB and, in turn, enhance their sensitivity to proteasome inhibitors both *in vitro* and *in vivo*. Bcl-2 is a direct transcription target of NF-κB and responsible for anti-apoptosis [30]. Our data showed that the anti-apoptotic Bcl-2 expression was induced by treatment with 1 μM lapatinib for three days in MDA-MB-231 cells and this effect was blocked by bortezomib (Figure 6A). Nevertheless, lapatinib and bortezomib in combination, but not individually, can induce pro-apoptotic Bax expression (Figure 6A) and enhance bortezomib-induced PARP and caspase-3 cleavages in MDA-MB-231 and HS-578 T cell lines, further demonstrating the synergistic effect of lapatinib on the proteasome inhibitor-induced apoptosis. In clonogenic assays, lapatinib and gefitinib did not reduce the viability of MDA-MB-231 and HS-578 T TNBC cell lines due to HER2-negative expression and the dispensable role of EGFR in these cells (Figure 6B). However, pretreatment with lapatinib but not gefitinib can enhance the anti-cancer activity of MG-132 and bortezomib (Figure 6B).

To further confirm this synergistic activity *in vivo*, MDA-MB-231 cells were injected into the mammary fat pad of SCID mice followed by treatment with lapatinib, bortezomib or lapatinib plus bortezomib, respectively. Treatment with bortezomib and lapatinib combined, but not individually, significantly suppressed the tumor growth (Figure 6C). The nuclear localization of p65 was enhanced in the lapatinib-treated tumors, and this effect was inhibited by treatment with bortezomib (Figure 6D). The combined treatment not only inhibited NF-κB but also induced Bax expression in the xenograft tumor tissues (Figure 6D). These results suggest that lapatinib may sensitize TNBC cells to proteasome inhibitors by increasing their oncogene addiction to NF-κB.

## Discussion

Unlike the luminal or HER2-enriched subtypes, characterized by the expression of hormone receptors or HER2, respectively, TNBC cells are almost insensitive to the established endocrine treatments and HER2-targeted agents due to the lack of known oncogenic drivers. Although synthetic lethal targeting of BRCA-deficient cells with PARP inhibitors has been proposed as a promising therapeutic strategy for TNBC in preclinical studies [31], the low mutation rate (near 20%) of BRCA1/2 [32] suggests that PARP inhibitors may only benefit a small number of such patients. New therapeutic strategies are therefore urgently needed for such patients.

Recent results from gene expression profiling analysis revealed that the NF-κB pathway may represent a key regulator of TNBC [33]. Various small molecules inhibiting NF-κB, including aspirin [34], genistein [35] and synthesized phospho-ibuprofen [36], have shown significant anti-tumor activity in preclinical studies for treating TNBC tumors. These studies revealed NF-κB as a potential therapeutic target for TNBC patients. Bortezomib (Velcade), a potent inhibitor of the 26S proteasome, has been approved for melanoma and hematopoietic malignancies. Preclinical studies have further demonstrated that bortezomib also showed remarkable anti-tumor activity against TNBC *in vitro*[37]. However, bortezomib alone [16] or in combination with aromatase inhibitor or tamoxifen [38] failed to show a significant clinical benefit in patients with metastatic breast cancer. The molecular heterogeneity and complexity of addicted status in TNBCs might explain these disappointing results [39]. Therefore, strategies to trap cancer cells to the NF-κB signal pathway by an artificial addiction shift may be able to potentiate the anti-tumor activity of proteasome inhibitors [40]. In this study, we reported that the off-target activity of lapatinib in augmenting NF-κB activity may enhance the therapeutic benefits of bortezomib for TNBC patients.

Induction of NF-κB activity was observed in lapatinib-treated HER2-positive breast cancer cells,and, thus, has been considered as a potential target for circumventing lapatinib resistance [19,20]. In addition to confirming the involvement of NF-κB activation in contributing to lapatinib resistance in HER2-positive breast cancer cells (Figure 5A), our data also showed that treatment with lapatinib elevates NF-κB activity in TNBC cell lines (Figure 1), uncovering the off-target effect of lapatinib on NF-κB activation. Lapatinib was tested as monotherapy or in combination with other systemic therapies in phase II trials for TNBC or HER2-negative breast cancers [41,42]. Inhibition of EGFR by lapatinib was considered as a promising therapeutic strategy for TNBC patients [43] since EGFR is overexpressed in 80% of TNBC [44]. Unfortunately, results from most of these studies showed limited clinical benefits for these patients. Nevertheless, the increased NF-κB activity renders lapatinib-treated TNBCs more vulnerable to NF-κB inhibition by p65 shRNA or proteasome inhibitors (Figure 5). These results strongly suggest that lapatinib may augment the oncogenic addiction of cancer cells to NF-κB, which may become the Achilles’ heel in TNBCs. The switch of survival pathway to NF-κB was also observed in various types of solid tumors during the acquisition of resistance to camptothecin and rendered these camptothecin-resistant cells more sensitive to the NF-κB inhibitor dehydroxymethylepoxyquinomicin (DHMEQ) [45]. Our results further demonstrated that co-treatment with lapatinib can sensitize TNBC cells to proteasome inhibitors both *in vitro* and *in vivo* (Figure 6), suggesting that the artificial trap of cancer cells to NF-κB signaling by lapatinib may be a potential strategy to increase the anti-tumor activity of bortezomib for TNBC patients.

Several lines of evidence from this study and the literature indicate that the induction of NF-κB by lapatinib is independent of EGFR and HER2 inhibition. First, regardless of the HER2 status, lapatinib-induced NF-κB activation was found in both HER2-positive and TNBC cells in this study (Figure 1A and B, respectively). Second, although lapatinib also possesses inhibitory activity against EGFR, our data showed that treatments with specific EGFR inhibitors, including erlotinib and gefitinib, suppress rather than induce p65 phosphorylations in both HER2-positive BT474 and triple-negative MDA-MB-231 breast cancer cells (Figure 1E and F). Also, only lapatinib but not gefitinib can synergize the anti-tumor activity of MG-132 in TNBC cells (Figure 6B). Third, inhibition of either HER2 [46] or EGFR [47] by its specific siRNA has been reported to decrease but not increase NF-κB activity. Similar to this EGFR/HER2-independent role in NF-κB activation, lapatinib has also been reported to up-regulate the expression of pro-apoptotic TRAIL death receptors DR4 and DR5 through an off-target mechanism in colon cancer cells and, thus, sensitizes these cancer cells to TRAIL-induced apoptosis [21]. The induction of DR5 by lapatinib was evident only with high drug concentrations (>5 μM) [21]. However, our data showed that 1 μM of lapatinib is sufficient for NF-κB activation. Therefore, lapatinib-enhanced NF-κB activity is unlikely through induction of DR5 expression although TRAIL has been known to initiate signaling to NF-κB activation [45].

The lapatinib-augmented NF-κB activity, derived from the EGFR/HER2-independent off target effect, involves both classic and SFK-mediated NF-κB activation pathways. Although our data revealed that reduction of the IκBα protein level was not accompanied with lapatinib-induced NF-κB activation, IκBα Ser32/36 phosphorylations and a higher turnover rate of IκBα were still found in lapatinib-treated cells, indicating that IκBα degradation remains necessary for liberating NF-κB. However, the *de novo* synthesis of IκBα, which is mediated by lapatinib-activated NF-κB, accounts for the unchanged IκBα protein level but did not feedback bind to and inhibit NF-κB. Up-regulation of SFK activity has been found in the acquired lapatinib-resistant cells with HER2 overexpression [48]. Our data further showed that lapatinib also activates SFK in both HER2-positive and TNBC cells to mediate IκBα Tyr42 phosphorylation, which prevents the feedback inhibition of NF-κB from IκBα protein binding [28,29]. These events lead to the constitutive activation of NF-κB in lapatinib-treated cells. Interestingly, bortezomib not only inhibits p65 phosphorylation but also reduces SFK tyrosine phosphorylation (data not shown). It suggests that the anti-tumor activity of bortezomib with co-treatment of lapatinib in our study may be partly attributed to inhibition of SFK activity by bortezomib. However, the potential possibility and molecular mechanisms underlying bortezomib-mediated SFK inhibition await further investigations.

## Conclusions

In conclusion, the off-target activity of lapatinib switches or enhances the oncogenic addiction of TNBC cells to NF-κB in a SFK-dependent manner. Therefore, co-treatment with lapatinib may synergize the anti-cancer activity of proteasome inhibitors, which may provide a novel and feasible way for treating TNBCs.

## Abbreviations

EGFR: Epidermal growth factor receptor; ELISA: Enzyme-linked immunosorbent assay; HER2/ERBB2: Epidermal growth factor receptor 2; HRP: Horseradish peroxidase; IKK: IκB kinase; IPA: Ingenuity pathway analysis; NF-κB/p65: Nuclear factor kappa-light-chain-enhancer of activated B cells; PARP: Poly ADP ribose polymerase; PBS: Phosphate-buffered saline; PCR: Polymerase chain reaction; PSI: Proteasome inhibitor I; SCID: Severe combined immunodeficient; SFK: Src family kinase; shRNA: Small hairpin RNA; siRNA: Small interfering RNA; TKI: Tyrosine kinase inhibitor; TNBC: Triple-negative breast cancer.

## Competing interests

The authors declare they have no competing interests.

## Authors’ contributions

YJC carried out the western blot analysis and drafted the manuscript. MHY participated in the design and coordination of the study and drafted the manuscript. MCY carried out the IPA analysis and immunoprecipitation assays, and was involved in drafting the manuscript. YLW, WSC and JYC participated in the animal studies and helped to revise the manuscript. CYS, YLY, CHC, CYT, PHC and TCH performed the immunoblot, immunohistochemical staining and reporter assays, and helped to revise the manuscript. SHL carried out the statistical analysis and drafted the manuscript. WCH conceived of the study, participated in the design of the study and contributed to the manuscript. All authors read and approved the final manuscript.

## Supplementary Material

Additional file 1 **Figure S1.** Lapatinib withdrawal reduced the Ser536 phosphorylation of p65 in lapatinib-selected clones. SkBr3/Lap#6, BT474/Lap#3, and 231/Lap#6 cells were cultured in the absence of lapatinib for one or three days, and total lysates were prepared and subjected to Western blot analysis with the indicated antibodies. **Figure S2.** Lapatinib does not induce the Ser276 and Ser529 phosphorylations of p65 in breast cancer cells. Whole cell lysates in SkBr3, BT474, MDA-MB-231 and their lapatinib-treated derivatives were harvested. Total lysates from SkBr3 (A) and MDA-MB-231 (B) cells treated with 10 ng/ml TNF-α for 10 minutes are used as a positive control. Phosphorylation of p65 at Ser276 (A-B) and Ser529 (C-D) was examined by Western blot analysis. **Figure S3.** The effect of lapatinib on the expression of *IL-1β* and *IL-6* transcripts in SkBr3, MDA-MB-231 cells, and their lapatinib-treated clones. A-B, SkBr3 and MDA-MB-231 cells were treated with lapatinib for 24 hours. C-D, SkBr3/Lap#6 and 231/Lap#2 cells were cultured in the presence or absence of lapatinib for 24 hours. Total RNA was extracted and subjected to RT-qPCR analysis for mRNA levels of *IL-1β* (A and C) and *IL-6* (B and D). **Figure S4.** The effect of lapatinib on the activation of SFK and IκBα Tyr42 phosphorylation in SkBr3, MDA-MB-231 cells, and their lapatinib-treated clones. A and C, BT474 (A) and MDA-MB-231 (C) cells were treated with 1 μM lapatinib for the indicated number of days. B, SkBr3/Lap#6 and 231/Lap#12 cells were cultured in the presence or absence of lapatinib for the indicated number of days. Total protein lysates were extracted and subjected to Western blot analysis with the indicated antibodies. Click here for file

Additional file 2: Table S1 Microarray analysis of upregulated gene expression profile in lapatinib-resistant SkBr3 and BT474 breast cancer cells. Click here for file

## References

[B1] Anders C Carey LA Understanding and treating triple-negative breast cancer Oncology (Williston Park) 2008 15 1233 1239 discussion 1239–1240, 1243 18980022 PMC2868264

[B2] Bertucci F Finetti P Cervera N Esterni B Hermitte F Viens P Birnbaum D How basal are triple-negative breast cancers? Int J Cancer 2008 15 236 240 10.1002/ijc.23518 18398844

[B3] Carey LA Perou CM Livasy CA Dressler LG Cowan D Conway K Karaca G Troester MA Tse CK Edmiston S Deming SL Geradts J Cheang MC Nielsen TO Moorman PG Earp HS Millikan RC Race, breast cancer subtypes, and survival in the Carolina Breast Cancer Study JAMA 2006 15 2492 2502 10.1001/jama.295.21.2492 16757721

[B4] Rouzier R Perou CM Symmans WF Ibrahim N Cristofanilli M Anderson K Hess KR Stec J Ayers M Wagner P Morandi P Fan C Rabiul I Ross JS Hortobagyi GN Pusztai L Breast cancer molecular subtypes respond differently to preoperative chemotherapy Clin Cancer Res 2005 15 5678 5685 10.1158/1078-0432.CCR-04-2421 16115903

[B5] Kassam F Enright K Dent R Dranitsaris G Myers J Flynn C Fralick M Kumar R Clemons M Survival outcomes for patients with metastatic triple-negative breast cancer: implications for clinical practice and trial design Clin Breast Cancer 2009 15 29 33 10.3816/CBC.2009.n.005 19299237

[B6] Lin NU Claus E Sohl J Razzak AR Arnaout A Winer EP Sites of distant recurrence and clinical outcomes in patients with metastatic triple-negative breast cancer: high incidence of central nervous system metastases Cancer 2008 15 2638 2645 10.1002/cncr.23930 18833576 PMC2835546

[B7] Pikarsky E Porat RM Stein I Abramovitch R Amit S Kasem S Gutkovich-Pyest E Urieli-Shoval S Galun E Ben-Neriah Y NF-kappaB functions as a tumour promoter in inflammation-associated cancer Nature 2004 15 461 466 10.1038/nature02924 15329734

[B8] Wang CY Mayo MW Baldwin AS Jr TNF- and cancer therapy-induced apoptosis: potentiation by inhibition of NF-kappaB Science 1996 15 784 787 10.1126/science.274.5288.784 8864119

[B9] Kanarek N London N Schueler-Furman O Ben-Neriah Y Ubiquitination and degradation of the inhibitors of NF-kappaB Cold Spring Harb Perspect Biol 2010 15 a000166 20182612 10.1101/cshperspect.a000166 PMC2828279

[B10] Sharma HW Narayanan R The NF-kappaB transcription factor in oncogenesis Anticancer Res 1996 15 589 596 8687102

[B11] Van Antwerp DJ Martin SJ Kafri T Green DR Verma IM Suppression of TNF-alpha-induced apoptosis by NF-kappaB Science 1996 15 787 789 10.1126/science.274.5288.787 8864120

[B12] Fan Y Dutta J Gupta N Fan G Gelinas C Regulation of programmed cell death by NF-kappaB and its role in tumorigenesis and therapy Adv Exp Med Biol 2008 15 223 250 10.1007/978-1-4020-6554-5_11 18437897

[B13] Orlowski RZ Kuhn DJ Proteasome inhibitors in cancer therapy: lessons from the first decade Clin Cancer Res 2008 15 1649 1657 10.1158/1078-0432.CCR-07-2218 18347166

[B14] Biswas DK Cruz AP Gansberger E Pardee AB Epidermal growth factor-induced nuclear factor kappa B activation: a major pathway of cell-cycle progression in estrogen-receptor negative breast cancer cells Proc Natl Acad Sci U S A 2000 15 8542 8547 10.1073/pnas.97.15.8542 10900013 PMC26984

[B15] Nakshatri H Bhat-Nakshatri P Martin DA Goulet RJ Jr Sledge GW Jr Constitutive activation of NF-kappaB during progression of breast cancer to hormone-independent growth Mol Cell Biol 1997 15 3629 3639 9199297 10.1128/mcb.17.7.3629 PMC232215

[B16] Yang CH Gonzalez-Angulo AM Reuben JM Booser DJ Pusztai L Krishnamurthy S Esseltine D Stec J Broglio KR Islam R Hortobagyi GN Cristofanilli M Bortezomib (VELCADE) in metastatic breast cancer: pharmacodynamics, biological effects, and prediction of clinical benefits Ann Oncol 2006 15 813 817 10.1093/annonc/mdj131 16403809

[B17] Geyer CE Forster J Lindquist D Chan S Romieu CG Pienkowski T Jagiello-Gruszfeld A Crown J Chan A Kaufman B Skarlos D Campone M Davidson N Berger M Oliva C Rubin SD Stein S Cameron D Lapatinib plus capecitabine for HER2-positive advanced breast cancer N Engl J Med 2006 15 2733 2743 10.1056/NEJMoa064320 17192538

[B18] Chen FL Xia W Spector NL Acquired resistance to small molecule ErbB2 tyrosine kinase inhibitors Clin Cancer Res 2008 15 6730 6734 10.1158/1078-0432.CCR-08-0581 18980964

[B19] Xia W Bacus S Hegde P Husain I Strum J Liu L Paulazzo G Lyass L Trusk P Hill J Harris J Spector NL A model of acquired autoresistance to a potent ErbB2 tyrosine kinase inhibitor and a therapeutic strategy to prevent its onset in breast cancer Proc Natl Acad Sci U S A 2006 15 7795 7800 10.1073/pnas.0602468103 16682622 PMC1472524

[B20] Xia W Bacus S Husain I Liu L Zhao S Liu Z Moseley MA 3rd Thompson JW Chen FL Koch KM Spector NL Resistance to ErbB2 tyrosine kinase inhibitors in breast cancer is mediated by calcium-dependent activation of RelA Mol Cancer Ther 2010 15 292 299 20124457 10.1158/1535-7163.MCT-09-1041

[B21] Dolloff NG Mayes PA Hart LS Dicker DT Humphreys R El-Deiry WS Off-target lapatinib activity sensitizes colon cancer cells through TRAIL death receptor up-regulation Sci Transl Med 2011 15 86ra50 21653830 10.1126/scitranslmed.3001384 PMC3769950

[B22] Hsia TC Tu CY Chen YJ Wei YL Yu MC Hsu SC Tsai SL Chen WS Yeh MH Yen CJ Yu YL Huang TC Huang CY Hung MC Huang WC Lapatinib-mediated COX-2 expression via EGFR/HuR interaction enhances the aggressiveness of triple-negative breast cancer cells Mol Pharmacol 2013 15 857 869 10.1124/mol.112.082743 23355539

[B23] Global gene expression changes in HER2-positive breast cancer cell lines in response to lapatinib resistance http://www.ncbi.nlm.nih.gov/geo/query/acc.cgi?acc=GSE51889

[B24] Inference of NF-kB-targeted gene expressions by Ingenuity Pathway Analysishttp://www.ingenuity.com

[B25] Sakurai H Chiba H Miyoshi H Sugita T Toriumi W IkappaB kinases phosphorylate NF-kappaB p65 subunit on serine 536 in the transactivation domain J Biol Chem 1999 15 30353 30356 10.1074/jbc.274.43.30353 10521409

[B26] Wang D Westerheide SD Hanson JL Baldwin AS Jr Tumor necrosis factor alpha-induced phosphorylation of RelA/p65 on Ser529 is controlled by casein kinase II J Biol Chem 2000 15 32592 32597 10938077 10.1074/jbc.M001358200

[B27] Zhong H Voll RE Ghosh S Phosphorylation of NF-kappa B p65 by PKA stimulates transcriptional activity by promoting a novel bivalent interaction with the coactivator CBP/p300 Mol Cell 1998 15 661 671 10.1016/S1097-2765(00)80066-0 9660950

[B28] Garg A Aggarwal BB Nuclear transcription factor-kappaB as a target for cancer drug development Leukemia 2002 15 1053 1068 10.1038/sj.leu.2402482 12040437

[B29] Imbert V Rupec RA Livolsi A Pahl HL Traenckner EB Mueller-Dieckmann C Farahifar D Rossi B Auberger P Baeuerle PA Peyron JF Tyrosine phosphorylation of I kappa B-alpha activates NF-kappa B without proteolytic degradation of I kappa B-alpha Cell 1996 15 787 798 10.1016/S0092-8674(00)80153-1 8797825

[B30] Vogler M BCL2A1: the underdog in the BCL2 family Cell Death Differ 2012 15 67 74 10.1038/cdd.2011.158 22075983 PMC3252829

[B31] Farmer H McCabe N Lord CJ Tutt AN Johnson DA Richardson TB Santarosa M Dillon KJ Hickson I Knights C Martin NM Jackson SP Smith GC Ashworth A Targeting the DNA repair defect in BRCA mutant cells as a therapeutic strategy Nature 2005 15 917 921 10.1038/nature03445 15829967

[B32] Gonzalez-Angulo AM Timms KM Liu S Chen H Litton JK Potter J Lanchbury JS Stemke-Hale K Hennessy BT Arun BK Hortobagyi GN Do KA Mills GB Meric-Bernstam F Incidence and outcome of BRCA mutations in unselected patients with triple receptor-negative breast cancer Clin Cancer Res 2011 15 1082 1089 10.1158/1078-0432.CCR-10-2560 21233401 PMC3048924

[B33] Ossovskaya V Wang Y Budoff A Xu Q Lituev A Potapova O Vansant G Monforte J Daraselia N Exploring molecular pathways of triple-negative breast cancer Genes Cancer 2011 15 870 879 10.1177/1947601911432496 22593799 PMC3352156

[B34] Chattopadhyay M Kodela R Nath N Barsegian A Boring D Kashfi K Hydrogen sulfide-releasing aspirin suppresses NF-kappaB signaling in estrogen receptor negative breast cancer cells in vitro and in vivo Biochem Pharmacol 2012 15 723 732 10.1016/j.bcp.2011.12.019 22209867

[B35] Pan H Zhou W He W Liu X Ding Q Ling L Zha X Wang S Genistein inhibits MDA-MB-231 triple-negative breast cancer cell growth by inhibiting NF-kappaB activity via the Notch-1 pathway Int J Mol Med 2012 15 337 343 22580499 10.3892/ijmm.2012.990

[B36] Sun Y Rowehl LM Huang L Mackenzie GG Vrankova K Komninou D Rigas B Phospho-ibuprofen (MDC-917) suppresses breast cancer growth: an effect controlled by the thioredoxin system Breast Cancer Res 2012 15 R20 10.1186/bcr3105 22293394 PMC3496138

[B37] Jones MD Liu JC Barthel TK Hussain S Lovria E Cheng D Schoonmaker JA Mulay S Ayers DC Bouxsein ML Stein GS Mukherjee S Lian JB A proteasome inhibitor, bortezomib, inhibits breast cancer growth and reduces osteolysis by downregulating metastatic genes Clin Cancer Res 2010 15 4978 4989 10.1158/1078-0432.CCR-09-3293 20843837 PMC2955762

[B38] Trinh XB Sas L Van Laere SJ Prove A Deleu I Rasschaert M Van de Velde H Vinken P Vermeulen PB Van Dam PA Wojtasik A De Mesmaeker P Tjalma WA Dirix LY A phase II study of the combination of endocrine treatment and bortezomib in patients with endocrine-resistant metastatic breast cancer Oncol Rep 2012 15 657 663 22134540 10.3892/or.2011.1562

[B39] Peddi PF Ellis MJ Ma C Molecular basis of triple negative breast cancer and implications for therapy Int J Breast Cancer 2012 15 217185 22295242 10.1155/2012/217185 PMC3262606

[B40] Togano T Sasaki M Watanabe M Nakashima M Tsuruo T Umezawa K Higashihara M Watanabe T Horie R Induction of oncogene addiction shift to NF-kappaB by camptothecin in solid tumor cells Biochem Biophys Res Commun 2009 15 60 64 10.1016/j.bbrc.2009.09.066 19778522

[B41] Burstein HJ Storniolo AM Franco S Forster J Stein S Rubin S Salazar VM Blackwell KL A phase II study of lapatinib monotherapy in chemotherapy-refractory HER2-positive and HER2-negative advanced or metastatic breast cancer Ann Oncol 2008 15 1068 1074 10.1093/annonc/mdm601 18283035

[B42] Boussen H Cristofanilli M Zaks T DeSilvio M Salazar V Spector N Phase II study to evaluate the efficacy and safety of neoadjuvant lapatinib plus paclitaxel in patients with inflammatory breast cancer J Clin Oncol 2010 15 3248 3255 10.1200/JCO.2009.21.8594 20530274

[B43] Dhillon N A Study of Lapatinib in Combination With Everolimus in Patients With Advanced, Triple Negative Breast Cancer (cited 2011 Feb 27). Available from: http://clinicaltrials.gov/show/NCT01272141 NLM Identifier: NCT01272141

[B44] Siziopikou KP Ariga R Proussaloglou KE Gattuso P Cobleigh M The challenging estrogen receptor-negative/ progesterone receptor-negative/HER-2-negative patient: a promising candidate for epidermal growth factor receptor-targeted therapy? Breast J 2006 15 360 362 10.1111/j.1075-122X.2006.00276.x 16848847

[B45] Tang W Wang W Zhang Y Liu S Liu Y Zheng D Tumour necrosis factor-related apoptosis-inducing ligand (TRAIL)-induced chemokine release in both TRAIL-resistant and TRAIL-sensitive cells via nuclear factor kappa B FEBS J 2009 15 581 593 10.1111/j.1742-4658.2008.06809.x 19120450

[B46] Merkhofer EC Cogswell P Baldwin AS Her2 activates NF-kappaB and induces invasion through the canonical pathway involving IKKalpha Oncogene 2010 15 1238 1248 10.1038/onc.2009.410 19946332 PMC2829103

[B47] Xu X Steere RR Fedorchuk CA Pang J Lee JY Lim JH Xu H Pan ZK Maggirwar SB Li JD Activation of epidermal growth factor receptor is required for NTHi-induced NF-kappaB-dependent inflammation PLoS One 2011 15 e28216 10.1371/journal.pone.0028216 22132240 PMC3223233

[B48] Rexer BN Ham AJ Rinehart C Hill S Granja-Ingram Nde M Gonzalez-Angulo AM Mills GB Dave B Chang JC Liebler DC Arteaga CL Phosphoproteomic mass spectrometry profiling links Src family kinases to escape from HER2 tyrosine kinase inhibition Oncogene 2011 15 4163 4174 10.1038/onc.2011.130 21499296 PMC3204390

