# Supplementary material for: Lapatinib–induced NF-kappaB activation sensitizes triple-negative breast cancer cells to proteasome inhibitors
Source: Breast Cancer Res. 2013 Nov 12;15(6):R108. doi: 10.1186/bcr3575 (PMC3979035; doi:10.1186/bcr3575)

Additional files

Additional file 1 –

Supplementary Figure S1. Lapatinib withdrawal reduced the Ser536 phosphorylation of p65 in lapatinib-selected clones. SkBr3/Lap#6, BT474/Lap#3, and 231/Lap#6 cells were cultured in the absence of lapatinib for 1or 3 days, and total lysates were prepared and subjected to Western blot analysis with indicated antibodies.

Supplementary Figure S1

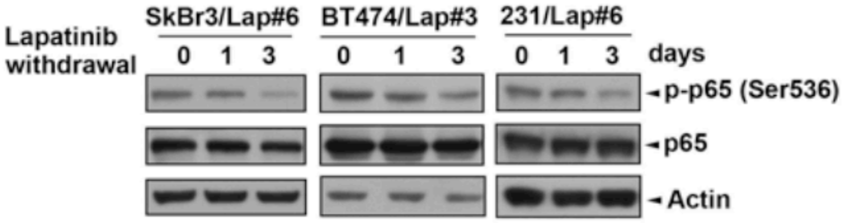

Supplementary Figure S2. Lapatinib dose not induce the Ser276 and Ser529 phosphorylations of p65 in breast cancer cells. Whole cell lysates in SkBr3, BT474, MDA-MB-231 and their lapatinib-treated derivatives were harvested. Total lysates from SkBr3 (A) and MDA-MB-231 (B) cells treated with 10 ng/ml TNF-α for 10 minutes are used as a positive control. Phosphorylation of p65 at Ser276 (A-B) and Ser529 (C-D) was examined by Western blot analysis.

Supplementary Figure S2

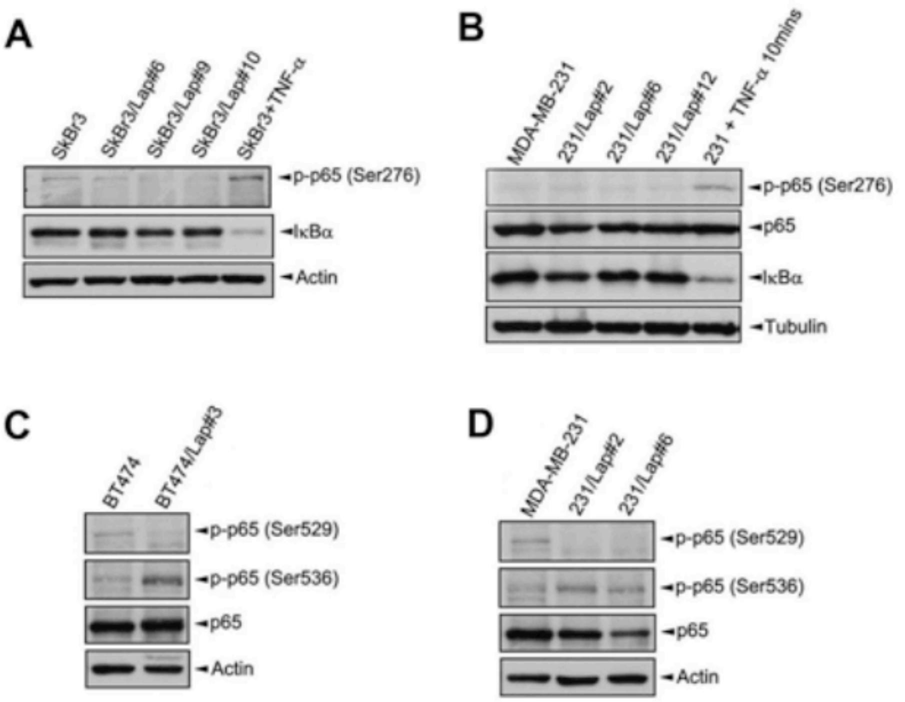

Supplementary Figure S3. The effect of lapatinib on the expression of *IL-1β* and *IL-6* transcripts in SkBr3, MDA-MB-231 cells, and their lapatinib-treated clones. **A-B,** SkBr3 and MDA-MB-231 cells were treated with lapatinib for 24 hours. **C-D,** SkBr3/Lap#6 and 231/Lap#2 cells were cultured in the presence or absence of lapatinib for 24 hours. Total RNA was extracted and subjected to RT-qPCR analysis for mRNA levels of *IL-1β* (A and C) and *IL-6* (B and D).

Supplementary Figure S3

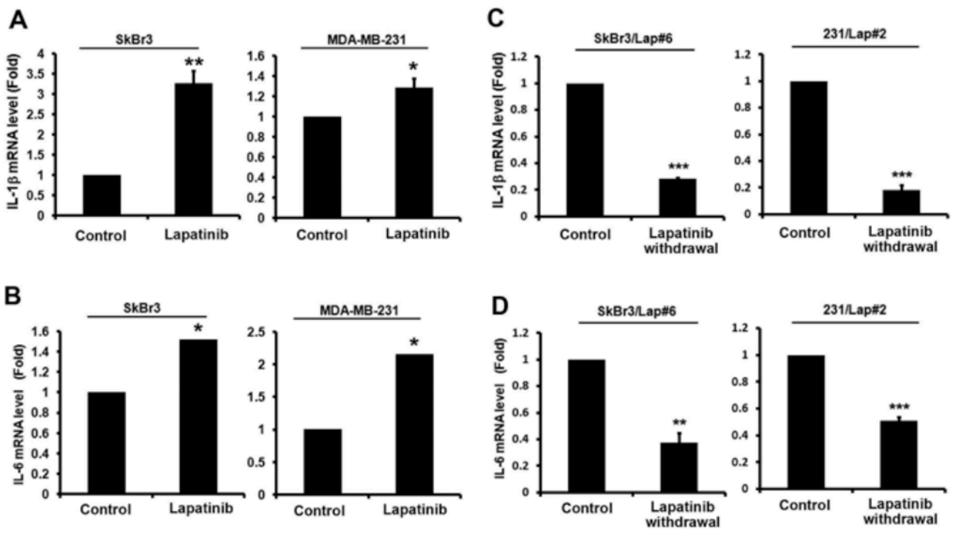

Supplementary Figure S4. The effect of lapatinib on the activation of SFK and IκBα Tyr42 phosphorylation in SkBr3, MDA-MB-231 cells, and their lapatinib-treated clones. **A and C,** BT474 (A) and MDA-MB-231 (C) cells were treated with 1 μM lapatinib for indicated days. **B,** SkBr3/Lap#6 and 231/Lap#12 cells were cultured in the presence or absence of lapatinib for indicated days. Total protein lysates were extracted and subjected to Western blot analysis with indicated antibodies.

Supplementary Figure S4

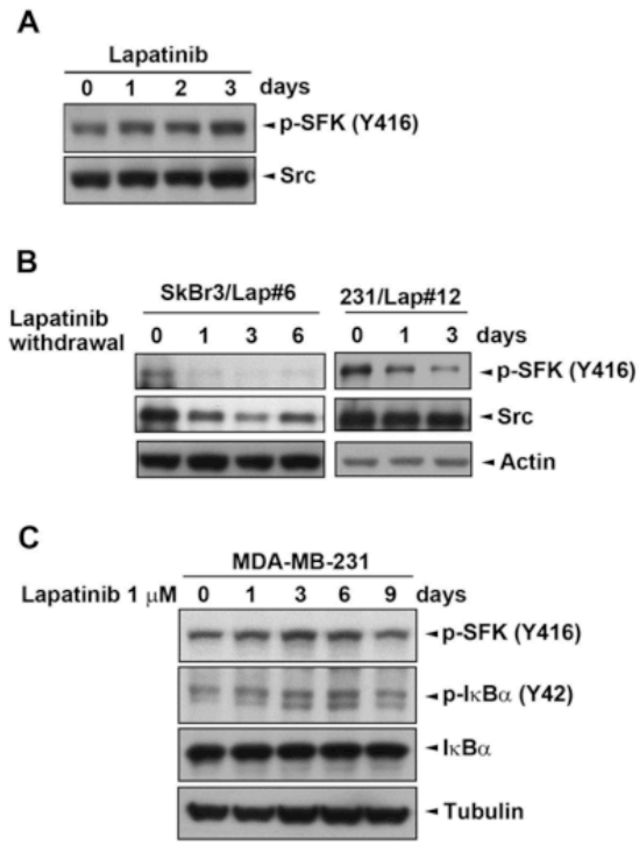

Supplement: Additional file 1 — Figure S1. Lapatinib withdrawal reduced the Ser536 phosphorylation of p65 in lapatinib-selected clones. SkBr3/Lap#6, BT474/Lap#3, and 231/Lap#6 cells were cultured in the absence of lapatinib for one or three days, and total lysates were prepared and subjected to Western blot analysis with the indicated antibodies. Figure S2. Lapatinib does not induce the Ser276 and Ser529 phosphorylations of p65 in breast cancer cells. Whole cell lysates in SkBr3, BT474, MDA-MB-231 and their lapatinib-treated derivatives were harvested. Total lysates from SkBr3 (A) and MDA-MB-231 (B) cells treated with 10 ng/ml TNF-α for 10 minutes are used as a positive control. Phosphorylation of p65 at Ser276 (A-B) and Ser529 (C-D) was examined by Western blot analysis. Figure S3. The effect of lapatinib on the expression of IL-1β and IL-6 transcripts in SkBr3, MDA-MB-231 cells, and their lapatinib-treated clones. A-B, SkBr3 and MDA-MB-231 cells were treated with lapatinib for 24 hours. C-D, SkBr3/Lap#6 and 231/Lap#2 cells were cultured in the presence or absence of lapatinib for 24 hours. Total RNA was extracted and subjected to RT-qPCR analysis for mRNA levels of IL-1β (A and C) and IL-6 (B and D). Figure S4. The effect of lapatinib on the activation of SFK and IκBα Tyr42 phosphorylation in SkBr3, MDA-MB-231 cells, and their lapatinib-treated clones. A and C, BT474 (A) and MDA-MB-231 (C) cells were treated with 1 μM lapatinib for the indicated number of days. B, SkBr3/Lap#6 and 231/Lap#12 cells were cultured in the presence or absence of lapatinib for the indicated number of days. Total protein lysates were extracted and subjected to Western blot analysis with the indicated antibodies. [file bcr3575-S1.pdf]
